# Job satisfaction, burnout, and safety behavior in air traffic controllers: a mediation analysis and decision tree insights

**DOI:** 10.3389/fpubh.2025.1626328

**Published:** 2025-09-12

**Authors:** Xiuyi Li, Zhuocheng Huang, Xing Peng, Junjie Zhang, Yueying Liu, Yuchuan Luo, Lingfeng Guo

**Affiliations:** ^1^CAAC Academy, Civil Aviation Flight University of China, Guanghan, Sichuan, China; ^2^School of Flight Technology, Civil Aviation Flight University of China, Guanghan, Sichuan, China; ^3^College of Aeronautical Engineering, Civil Aviation Flight University of China, Guanghan, Sichuan, China

**Keywords:** air traffic controllers, mediating effect, aviation safety, decision tree model, safety behavior

## Abstract

**Introduction:**

This study aims to explore the mediating role of burnout in the relationship between job satisfaction and safety behavior among air traffic controllers (ATCs), and to identify the most influential factor affecting ATCs’ safety behavior using a decision tree model.

**Methods:**

Data were collected from 357 ATCs using established questionnaires measuring job satisfaction, burnout, and safety behavior. Bootstrap analysis was employed to examine the mediating effect of burnout, and a decision tree model was applied to determine the key factors influencing safety behavior.

**Results:**

The bootstrap analysis revealed that burnout partially mediated the relationship between job satisfaction and safety behavior, accounting for 40% of the total effect. The decision tree model identified burnout as the primary predictor of safety behavior, followed by job satisfaction and other factors.

**Discussion:**

These findings underscore the critical role of burnout in influencing ATCs’ safety performance. Enhancing safety behavior should prioritize targeted interventions to reduce burnout, in addition to addressing job satisfaction and other contributing factors.

## 1 Introduction

Safety is the most paramount concern in aviation, and air traffic controllers (ATCs) play a critical role in ensuring this by coordinating the heading and altitude of aircraft. However, with the rapid development of China’s aviation industry, ATCs are under increasing pressure to ensure flight safety. Their safety behavior is influenced by a myriad of factors, including personality traits and environmental factors. Emotional stability (1), stress resilience (2), and attitude toward operations (3) are among the personal traits impacting their performance. Environmental factors such as safety-related stress and fatigue (4), job satisfaction (5), and burnout (6) also play critical roles.

Job satisfaction is defined as an emotional state resulting from the appraisal of one’s job (7). Staff members in the aviation industry, such as air traffic controllers and flight crew, exhibit different levels of dissatisfaction with their current jobs (5, 8). Higher job satisfaction among ATCs is associated with greater engagement and patience in their roles, potentially enhancing safety behavior (9). Positive correlations between job satisfaction and safety behavior have been observed in other high-stress occupations such as nursing and construction (10, 11). Accordingly, we hypothesize that job satisfaction positively affects ATCs’ safety behavior.

Burnout is a long-term response to chronic emotional and interpersonal stress at work (12). Previous studies have shown that burnout is a common phenomenon among employees in the aviation industry, such as pilots (6, 13), and ATCs (14). It often comes with negative effects, such as feeling tired after work and being pessimistic about one’s occupational future. These feelings may lead to a lack of motivation at work and a reduction in safety behavior among ATCs (15). The negative relationship between burnout and safety behavior can also be observed in other groups with high safety awareness demands, such as construction workers (16), firefighters (17), and food service employees (18). Thus, we hypothesize that ATC burnout negatively affects safety behavior.

Numerous prior studies have endeavored to ascertain the relationship between burnout and job satisfaction. These investigations have uncovered notable inverse correlations between job satisfaction and burnout among hospital staff (20), educators in schools (19), and flight crews (8). Declining levels of job satisfaction are often linked to the deterioration of interpersonal relationships and the absence of personal fulfillment from work, which ultimately lead to burnout. Therefore, we hypothesize that job satisfaction has a negative impact on the burnout of ATCs.

In conclusion, investigating the relationship between job satisfaction, burnout, and safety behavior among ATCs is imperative for three critical reasons. Firstly, as primary guardians of aviation safety, ATCs’ operational performance directly impacts millions of lives, yet they face escalating pressures from industry expansion. Secondly, empirical evidence confirms that job satisfaction enhances safety behavior through increased engagement (10, 11), while burnout induces safety-compromising behaviors via emotional exhaustion and demotivation (16, 17). Thirdly, existing research identifies burnout as a mediator between job satisfaction and job performance (21), we extend this model to hypothesize a mediating role of burnout in the relationship between job satisfaction and safety behavior among ATCs.

**Figure 1:**
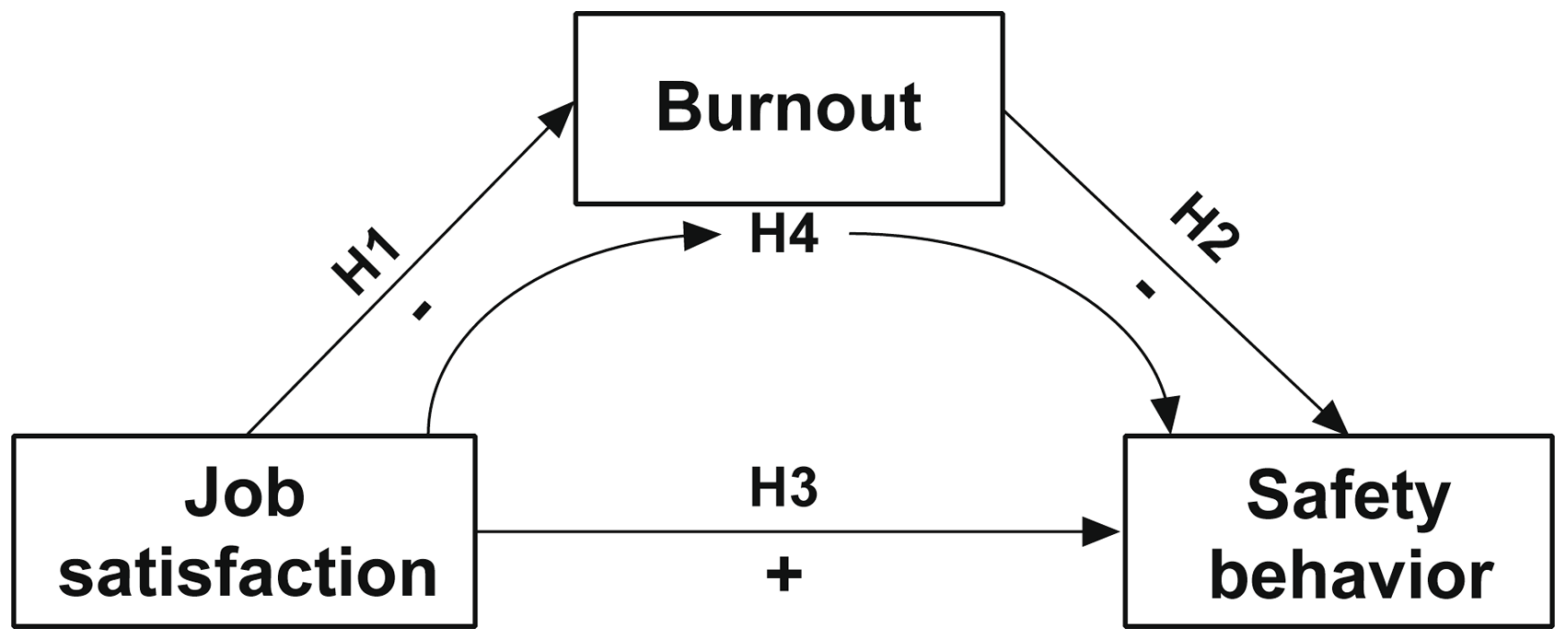
The hypothesis relationship between job satisfaction burnout and safety behavior among ATCs.

Based on the existing literature, we propose the following hypotheses, depicted in Figure 1.

*H*1: ATCs’ job satisfaction negatively affects burnout.

*H*2: ATCs’ burnout negatively affects their safety behavior.

*H*3: ATCs’ job satisfaction positively affects safety behavior.

*H*4: ATCs’ job satisfaction affects safety behavior through the mediation of burnout.

With the advancement of machine learning technologies, Feature Importance Analysis, as a core technique in machine learning, is reshaping traditional paradigms of variable selection and theoretical construction in psychology (22). Yarkoni provides a detailed discussion of how machine learning approaches, including feature importance analysis, are driving psychology’s shift from traditional explanatory paradigms toward predictive frameworks, thereby reshaping practices in variable selection and theory construction (22). In the realm of psychology, decision tree-based feature importance ranking has been effectively employed to pinpoint the most crucial influencing factors. For example, Li devised a job satisfaction analysis model and showed that the random forest algorithm founded on decision trees exhibited superior performance in terms of model accuracy and efficacy (23). Likewise, Giorgi constructed a recursive partitioning model to forecast perceived organizational support from multiple workplace characteristics, and identified two predominant influencing factors (24). The safety behavior of ATCs is influenced by a multitude of factors; however, there is still no conclusive evidence regarding which factors have the most significant impact. Consequently, another research question of this study is to explore the most influential factor affecting the safety behavior of ATCs.

## 2 Materials and methods

This study employed a questionnaire to collect data from ATCs. The comprehensive questionnaire consisted of four parts: (1) basic demographic information, (2) a job satisfaction survey, (3) a safety behavior assessment, and (4) a burnout questionnaire. Incomplete or abnormal responses were considered as invalid. Out of the 367 ATCs who participated in the study, 357 valid questionnaires were successfully collected. The participants voluntarily engaged in this research, and no illegal or unethical information was gathered. Each participant was rewarded upon completion of the questionnaire. Prior to participation in the online questionnaire survey, all subjects were delivered an electronic informed consent form detailing the study’s objectives, nature, and procedures. Only after voluntarily acknowledging acceptance of the electronic consent document did participants formally commence the study. This study was approved by the Ethics Committee of Civil Aviation Flight University of China (Ethics Committee Reference Number: CAFUC-2024审5号) and was performed in accordance with the approved guidelines and the Declaration of Helsinki.

### 2.1 Job satisfaction

The ATCs’ job satisfaction was assessed using a 14-item questionnaire revised by Zhou, including five dimensions. It was scored on a 5-point Likert scale ranging from 1 (strongly disapprove) to 5 (strongly approve). The five dimensions are the job itself, rewards, work environment, workgroups, and the whole enterprise. The job itself refers to the ATCs’ evaluation of the attractiveness, challenges, and whether the job utilizes their abilities. Rewards refer to the degree to which the ATCs acknowledge their compensation. Work environment refers to whether employees are satisfied with the working environment, which includes logistics, accommodation, equipment, and facilities provided by the organization for employees to carry out their work. Workgroups refer to the evaluation of co-worker relations and division of responsibilities in departments and posts. Whole enterprise refers to ATCs’ evaluation of management systems, corporate culture, and organizational structure. In the initial study, Cronbach’s *α* was 0.94, indicating high reliability (25). In the current study, Cronbach’s *α* of the ATCs’ job satisfaction questionnaire was 0.931. For the present sample, descriptive statistics of the measure are presented in Table 1.

**Table 1:** Description statistics.

Division	Variables	Manipulation of variables	*M*(*SD*)
Independent	Job satisfaction 3.26(0.78)	1. Overall job satisfaction level as an air traffic controller.	3.69(0.89)
		2. Perceived intensity of current workload.	3.43(1.11)
		3. Perception of work’s alignment with self-actualization needs.	3.39(1.08)
		4. Perceived equity between job contribution and compensation.	2.79(1.17)
		5. Availability of professional development opportunities.	3.07(1.06)
		6. Fairness and transparency in promotion mechanisms.	3.08(1.14)
		7. Adequacy of operational equipment and resources.	3.41(1.01)
		8. Organizational provision of restorative break facilities.	3.21(1.07)
		9. Satisfaction with organizational support services (e.g., catering, transportation).	3.13(1.11)
		10. Evaluation of colleague relationship quality.	3.96(0.86)
		11. Psychological safety in leader-subordinate communication.	3.31(1.01)
		12. Perceived rationality of management systems.	3.21(1.04)
		13. Assessment of organizational culture’s inclusivity.	2.96(1.15)
		14. Career development prospects in air traffic control.	2.99(1.13)
Mediating	Burnout 2.31(0.92)	1. Sistent physical and mental exhaustion attributable to work.	2.73(1.36)
		2. Daily depletion of energy reserves by the conclusion of work shifts.	2.82(1.43)
		3. Morning fatigue preceding workday commencement.	2.54(1.52)
		4. Chronic stress exposure throughout daily occupational activities.	2.47(1.45)
		5. Work-induced perception of psychological and physical depletion.	1.69(1.37)
		6. Progressive disengagement from core job responsibilities.	2.05(1.56)
		7. Diminishing intrinsic motivation toward occupational tasks.	2.26(1.61)
		8. Existential questioning regarding work’s significance.	1.67(1.61)
		9. Declining investment in work-related contributions.	1.94(1.64)
		10. Demonstrated efficacy in workplace problem resolution.	2.39(1.50)
		11. Perceived value-added impact on organizational outcomes.	2.42(1.66)
		12. Positive self-assessment of job-specific competencies.	2.12(1.54)
		13. Experienced accomplishment upon task completion.	2.63(1.59)
		14. Accumulation of substantively impactful work deliverables.	2.74(1.50)
		15. Confidence in efficient work product execution.	2.22(1.49)
Dependent	Safety behavior 4.12(0.68)	1. Consistent utilization of mandated personal protective equipment.	4.26(0.84)
		2. Strict adherence to established safety protocols during task execution.	4.42(0.74)
		3. Implementation of optimal hazard control measures in operations.	4.34(0.77)
		4. Active advocacy for organizational safety initiatives.	4.10(0.81)
		5. Expenditure of discretionary effort toward safety enhancement.	3.85(0.92)
		6. Voluntary engagement in safety improvement activities beyond formal requirements.	3.76(1.09)

### 2.2 Safety behavior

Griffin and Neal (26) differentiate safety behavior into two categories: safety compliance and safety participation. Safety compliance encompasses core activities such as adhering to safety regulations and using protective equipment to ensure personal and workplace safety. In contrast, safety participation involves behaviors like reporting potential hazards and engaging in safety training to foster a safety-supportive environment. This questionnaire consists of 6 items divided into these 2 dimensions, and responses are recorded on a 5-point Likert scale ranging from 1 (strongly disapprove) to 5 (strongly approve), primarily measuring employees’ active and passive involvement in work safety behaviors. In the initial study, Cronbach’s *α* for pilot’s safety behavior was 0.96, indicating high reliability (27). In the current study, Cronbach’s *α* for ATCs’ safety behavior in the questionnaire is 0.877. Descriptive statistics for the measure in the present sample are provided in Table 1.

### 2.3 Burnout

Maslach and Jackson’s MBI-General Survey (28), widely used across various occupations, comprises 15 items distributed across 3 dimensions: emotional exhaustion, depersonalization, and personal accomplishment. Emotional exhaustion refers to a state of overexertion of emotional resources, fatigue, and loss of energy at work. Depersonalization refers to a negative attitude and mood of indifference, detachment, or resignation toward work. Personal accomplishment refers to the positive feelings an individual has regarding their ability to perform the job effectively, maintaining enthusiasm and motivation. Low personal accomplishment is manifested as feelings of difficulty in performing the job, loss of enthusiasm and motivation, and a sense of powerlessness, depression, and lack of willingness to put effort into one’s work. Responses were recorded on a 7-point Likert scale ranging from 0 (never) to 6 (very often). Higher scores of emotional exhaustion and depersonalization mean more severe burnout, while lower scores on the personal accomplishment dimension mean higher levels of burnout. In a previous study on Chinese civil pilots, Cronbach’s *α* was 0.964 (29). In the current study, Cronbach’s *α* was 0.878. For the present sample, descriptive statistics of the measure are presented in Table 1.

### 2.4 Mediation effect analysis

IBM SPSS Statistics 28 was employed to compute the descriptive statistics, the correlations among the tested variables, and Hierarchical Multiple Regression. The Hierarchical Multiple Regression analysis incorporated ATC’s safety behavior. Three models were constructed. The dependent variable for all models is safety behavior. Model 1 includes demographic characteristics as independent variables; Model 2 incorporates demographic characteristics and subscales of job satisfaction; Model 3 comprises demographic characteristics, subscales of the job satisfaction scale, and subscales of the burnout scale. Bootstrapping, which is particularly useful for small or non-normally distributed samples, was used to confirm the mediating effect. Preacher and Hayes formally established bootstrapping as a statistically robust alternative to traditional methods. Their simulation studies demonstrated that bootstrapping provides more accurate confidence intervals for indirect effects, especially in small-to-moderate samples (30). Bias-corrected and accelerated 95% confidence intervals (95%*CI*) for direct and indirect effects were computed in Model 4 of PROCESS. Evidence for burnout’s mediating role was established when the 95% CI excluded 0.

### 2.5 Feature importance analysis

Building on our mediation analysis of how job satisfaction and burnout affect ATCs’ safety behavior, this study ranks and quantifies the importance of key influencing factors. To achieve this, we employed the widely-used decision tree model (31, 32) for feature importance ranking, where a variable’s importance is determined by its frequency of use in splitting nodes, weighted by the number of samples affected at each split, resulting in a higher importance score for features exerting greater influence on predictions. Focusing specifically on ranking continuous variables, we utilized the reduction in Mean Squared Error (*MSE*) as our evaluation metric within the decision tree algorithm. To do this, the algorithm calculates the change in MSE before and after the regression tree is split into two child nodes when feature *X_j_* is included:


$$\text{ΔMSE}={\mathrm{MSE}}_{t}-(\frac{N_{\mathrm{tL}}}{N_{t}}\mathrm{MS}E_{\mathrm{tL}}+\frac{N_{\mathrm{tR}}}{N_{t}}\mathrm{MS}E_{\mathrm{tR}})$$

Where *MSE_t_* was the *MSE* of the node before splitting, *MSE_tL_* and *MSE_tR_* were the *MSEs* of the left child node and the right child node after splitting, *N_t_*, *N_tL_*, and *N_tR_* were the number of samples before splitting, the number of samples in the left child node, and the number of samples in the right child node, respectively. The total importance of a feature (*X*) is then the sum of the Δ*MSE* values from all nodes where it was used for splitting; crucially, a larger summed Δ*MSE* directly indicates greater feature importance in predicting safety behavior.

This study employed a decision tree regression model to construct a predictive framework by using Python. Its core advantage lies in providing explicit feature importance metrics, offering direct empirical evidence for analyzing the influencing factors of ATCs’ safety behaviors. Model optimization was implemented through a systematic hyperparameter tuning procedure. This involved defining a grid search space encompassing nine key parameters: maximum depth (None, 5, 7, 9, 11, 15); maximum leaf nodes (None, 20, 40, 60), splitting control parameters: minimum samples split (0.01 to 0.1); minimum samples leaf (0.005 to 0.05), splitting strategy parameters: splitting criterion (squared_error, friedman_mse, absolute_error); splitting method (best, random), regularization parameters: complexity parameter (0.0 to 0.02); feature sampling fraction (sqrt, log2, 0.7, 0.8, None). Parameter optimization was executed via an efficient grid search framework. This strategy utilized 100 sampled parameter combinations to explore the high-dimensional parameter space, with parameter stability evaluated via 5-fold cross-validation.

## 3 Results

### 3.1 Demographic characteristics of ATCs

The demographic characteristics of the ATCs are shown in Table 2. The mean age was 30.5 ± 4.8 years. More than half of the ATCs were married (56.6%), and over one-third had worked for less than 5 years (35.3%), followed by those who had worked for 5–10 years (33.6%), and more than 10 years (31.1%). Most ATCs are frontline command (88.2%) and work at tower control (92.4%). Otherwise, Table 2 displays the safety behavior scores among ATCs with different demographic characteristics. The safety behavior scores of most of these groups were higher than 4, indicating that most ATCs performed highly safe practices at work. Results revealed statistical differences in position, ATCs who work at tower control have higher scores than area control in safety behavior.

**Table 2:** Demographic characteristics of ATCs.

Demographic characteristics	Groups	*N* = 357	%	Safety behavior *M*(*SD*)	*t*/*F*
Age	Under 24	46	12.9	4.12(0.85)	0.122
	25–29	128	35.9	4.10(0.60)	
	30–34	107	30.0	4.14(0.70)	
	Over 35	76	21.2	4.15(0.69)	
Working year	Under 5 years	126	35.3	4.13(0.73)	0.015
	6–10 years	120	33.6	4.12(0.58)	
	Over 11 years	111	31.1	4.11(0.74)	
Marital status	Married	202	56.6	4.14(0.68)	0.459
	Single	155	43.4	4.10(0.69)	
Post	Area control	27	7.6	3.80(1.00)	−2.610^**^
	Tower Control	330	92.4	4.15(0.64)	
Position	Leader	30	8.4	4.29(0.83)	2.633
	Operational Guidance	12	3.4	3.76(0.86)	
	Frontline command	315	88.2	4.12(0.66)	

**p<0.01.

### 3.2 Correlations among job satisfaction, burnout, and safety behavior

Table 3 shows the correlations for ATCs’ job satisfaction, burnout, and safety behavior and their dimensions. The results showed that Job satisfaction and its dimensions showed significant negative correlations with burnout and its dimensions (*p* < 0.01). Burnout and its dimensions were significantly negatively correlated with safety behavior and its dimensions (*p* < 0.01). Otherwise, job satisfaction and its dimensions demonstrated significant positive correlations with safety behavior and its dimensions (*p* < 0.01).

**Table 3:** Intercorrelations among observed variables (*N* = 357).

Variables	1	2	3	4	5	6	7	8	9	10	11	12	13
WI (1)	1												
REW (2)	0.650^**^	1											
WEV (3)	0.507^**^	0.675^**^	1										
WG (4)	0.521^**^	0.593^**^	0.563^**^	1									
WEP (5)	0.667^**^	0.739^**^	0.638^**^	0.649^**^	1								
JS (6)	0.795^**^	0.888^**^	0.816^**^	0.763^**^	0.896^**^	1							
EX (7)	−0.472^**^	−0.416^**^	−0.342^**^	−0.328^**^	−0.472^**^	−0.497^**^	1						
DEP (8)	−0.522^**^	−0.413^**^	−0.302^**^	−0.452^**^	−0.475^**^	−0.519^**^	0.635^**^	1					
PA (9)	−0.187^**^	−0.196^**^	−0.209^**^	−0.214^**^	−0.183^**^	−0.236^**^	0.050	0.166^**^	1				
BN (10)	−0.531^**^	−0.465^**^	−0.392^**^	−0.451^**^	−0.510^**^	−0.568^**^	0.746^**^	0.795^**^	0.621^**^	1			
SC (11)	0.287^**^	0.240^**^	0.248^**^	0.324^**^	0.214^**^	0.317^**^	−0.156^**^	−0.345^**^	−0.292^**^	−0.370^**^	1		
SP (12)	0.378^**^	0.357^**^	0.369^**^	0.352^**^	0.387^**^	0.448^**^	−0.281^**^	−0.399^**^	−0.264^**^	−0.434^**^	0.564^**^	1	
SB (13)	0.380^**^	0.342^**^	0.354^**^	0.383^**^	0.347^**^	0.438^**^	−0.253^**^	−0.423^**^	−0.313^**^	−0.457^**^	0.865^**^	0.902^**^	1

**p<0.01. WI, work itself; REW, reward; WEV, working environment; WG, workgroups; WEP, whole enterprise; JS, job satisfaction; EX, emotional exhaustion; DEP, Depersonalization; PA, accomplishment; BN, burnout; SC, safety compliance; SP, safety participation; SB, safety behavior.

### 3.3 Regression analysis of job satisfaction, burnout, and safety behavior

Table 4 presents the results of the hierarchical multiple regression models for safety behavior. Model 1 explained a small but significant portion of the variance in safety behavior (*R*^2^ = 0.024). Within this block, only position emerged as a significant positive predictor (*β* = 0.139, *p*<0.05). Model 2 significantly improved the prediction of safety behavior (*R*^2^ = 0.218). Work itself (*β* = 0.211, *p* < 0.01), work environment (*β* = 0.153, *p* < 0.05), and workgroups (*β* = 0.185, *p* < 0.05) were significant positive predictors of safety behavior at this stage. Model 3 provided a further significant increase in explanatory power (*R*^2^ = 0.308). Emotional exhaustion (*β* = −0.308, *p* < 0.001) and personal accomplishment (*β* = −0.185, *p* < 0.001) emerged as strong negative predictors of safety behavior. Work environment remained a significant positive predictor (*β* = 0.164, *p* < 0.05).

**Table 4:** The hierarchical multiple regression models of safety behavior.

Dependent variables	Safety behavior					
	Model 1		Model 2		Model 3	
	*β*	95%*CI*	*β*	95%*CI*	*β*	95%*CI*
BLOCK 1: Demographic characteristics and work-related characteristics						
Age	0.075	[−0.077, 0.183]	0.072	[−0.067, 0.169]	0.077	[−0.057, 0.166]
Working year	−0.095	[−0.207, 0.067]	−0.062	[−0.170, 0.078]	−0.078	[−0.175, 0.060]
Marital status	0.020	[−0.206, 0.153]	−0.034	[−0.209, 0.1160]	−0.040	[−0.208, 0.100]
Position	0.139^*^	[0.089, 0.625]	0.108	[0.034, 0.522]	0.094	[0.012, 0.475]
Post	−0.056	[−0.201, 0.068]	−0.019	[−0.145, 0.100]	−0.002	[−0.118, 0.114]
BLOCK 2: Job satisfaction						
Work itself			0.211^**^	[0.055, 0.247]	0.111	[−0.015, 0.175]
Reward			0.010	[−0.104, 0.118]	0.004	[−0.103, 0.108]
Work environment			0.153^*^	[0.013, 0.224]	0.164^*^	[0.026, 0.228]
Workgroups			0.185^*^	[0.044, 0.250]	0.095	[−0.025, 0.175]
Whole enterprise			−0.024	[−0.129, 0.096]	−0.045	[−0.139, 0.076]
BLOCK 3: Burnout						
Depersonalization					0.073	[−0.027, 0.105]
Emotional exhaustion					−0.308^***^	[−0.204, −0.085]
Personal accomplishment					−0.185^***^	[−0.155, −0.052]
*R* ^2^	0.024		0.218		0.308	
Δ*R*^2^	0.024		0.194		0.090	

*p<0.05, **p<0.01, ***p<0.001.

### 3.4 The mediating effect of burnout on the relationship of job satisfaction and safety behavior

The results of the hierarchical regression analysis for PROCESS Model 4 are presented in Table 5. Job satisfaction significantly and negatively predicted job burnout (*β* = −0.677, *p* < 0.001), which supported H1. Subsequently, job satisfaction significantly and positively predicted safety behavior (*β* = 0.385, *p* < 0.001), supporting H3. Finally, when both job burnout and job satisfaction were included simultaneously in the regression model, job satisfaction positively predicted safety behavior (*β* = 0.231, *p* < 0.001), while job burnout negatively predicted safety behavior (*β* = −0.227, *p* < 0.001), supporting H2. Notably, job satisfaction retained a significant direct predictive effect on safety behavior, indicating that its indirect effect on safety behavior was also significant.

**Table 5:** Mediation effect analysis of burnout.

Path	*β*	*se*	*t*	*p*	95%*CI*
Job satisfaction Burnout	−0.677	0.052	−12.995	<0.001	[−0.780, −0.575]
Job satisfaction Safety behavior [Total effect]	0.385	0.042	9.177	<0.001	[0.303, 0.468]
Job satisfaction Safety behavior [Direct effect]	0.231	0.049	4.724	<0.001	[0.135, 0.328]
Burnout Safety behavior	−0.227	0.041	−5.527	<0.001	[−0.308, −0.146]
Job satisfaction Safety behavior [Indirect effect]	0.154	0.035	-	-	[0.087, 0.225]

**Figure 2:**
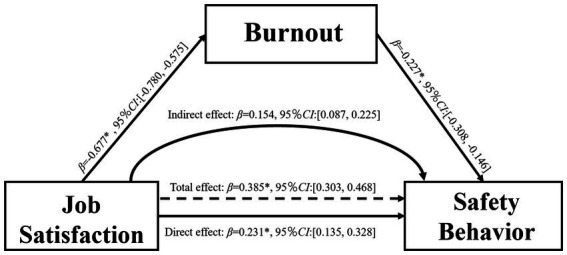
The mediating effect burnout in the relationship between job satisfaction and safety behavior.

Further examination of the indirect effect using the Bootstrap method (5,000 resamples, bias-corrected) is summarized in Table 5. The results revealed that the indirect effect of job satisfaction on safety behavior via job burnout was 0.154, the 95% bias-corrected confidence interval [0.087, 0.225] did not include zero, indicating a statistically significant indirect effect of job burnout. The total effect was 0.385 (95%*CI*: [0.303, 0.468]), and the direct effect was 0.231 (95%*CI*: [0.135, 0.328]). The indirect effect accounted for 40% of the total effect. The relationship among job satisfaction, burnout and safety behavior is shown in Figure 2, which supported H4.

### 3.5 Feature importance analysis of ATCs’ safety behavior

To further investigate the key factors’ contribution in ATCs’ safety behavior, this study employed the Z-score method (threshold = 3) to detect outliers in the numerical of ATC’s job satisfaction, burnout, and safety behavior. This process yielded a diagnostic report detailing the mean, standard deviation, and outlier distribution for each variable. Subsequently, a conservative cleaning strategy was implemented: samples were only removed if identified as outliers across at least two features, the final cleaned dataset retained 352 samples. Furthermore, a spearman correlation analysis was conducted among the variables in this study. The results revealed a strong positive correlation between working years and age (*r* = 0.819, *p* < 0.001). Consequently, to mitigate multicollinearity concerns, the age variable was excluded from the final predictive model. The retained predictors comprise job satisfaction, burnout, marital status, position, post, and working years.

**Figure 3:**
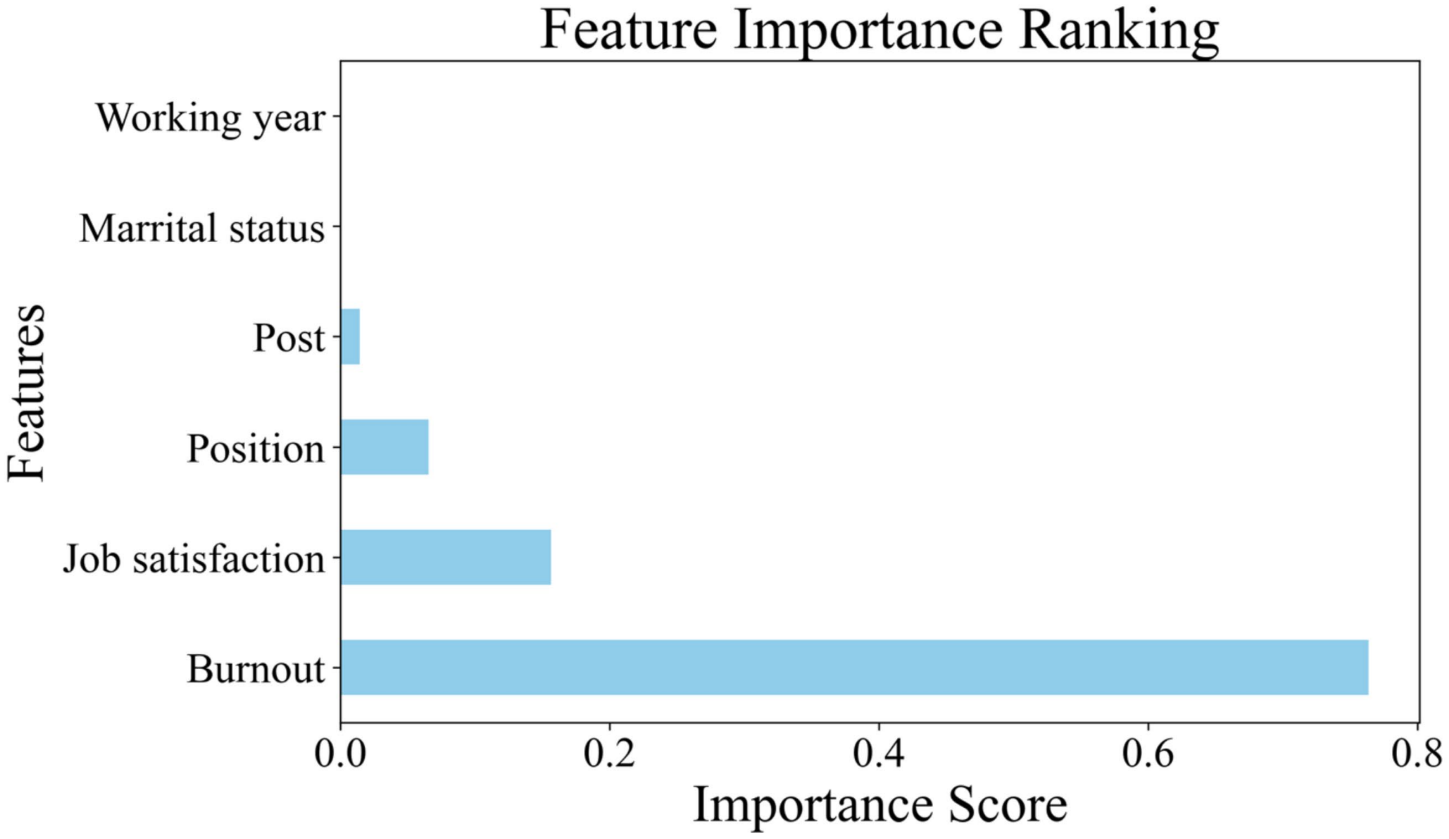
Feature importance analysis of ATCs’ safety behavior.

In this decision tree model, the optimized hyperparameter configuration was determined as follows: splitter = best, min samples split = 0.01, min samples leaf = 0.05, max leaf nodes = 60, max features = sqrt, max depth = 7, criterion = friedman mse, and ccp alpha = 0. This configuration achieved *R^2^* of 0.28 and *MSE* of 0.357on the test set, *R^2^* of 0.27 and *MSE* of 0.248 on the training set. As illustrated in Figure 3, the feature importance analysis of the final model identified burnout as the primary determinant of ATC safety behavior with a relative importance score of 0.763, followed by job satisfaction with a relative importance score of 0.156. Position and post demonstrated secondary predictive contributions with a relative importance score of 0.066 and 0.015, respectively. Notably, marital status and working years exhibited zero relative importance score, suggesting these variables lack significant influence on safety behavior outcomes.

## 4 Discussion

This research delves into two research inquiries: (1) whether burnout acts as a mediating variable in the association between job satisfaction and safety behaviors among air traffic controllers (ATCs); (2) which factors exert a significant impact on the safety behaviors of ATCs. By comprehensively assessing the mediating effect of burnout between the job satisfaction and safety behaviors of ATCs and leveraging a decision - tree model to analyze the feature significance of influencing factors on safety behaviors, this study addresses the lacuna in the extant literature concerning safety behavior research within the ATC domain.

### 4.1 The mediating effect of burnout on the relationship of job satisfaction and safety behavior

To address Research Question 1, this research performed hierarchical regression analysis to examine the effects of burnout and job satisfaction on safety behaviors, followed by bootstrap using PROCESS to validate the mediating effect.

As shown in Figure 2, job satisfaction had a negative influence on burnout, thereby validating Hypothesis H1. This finding resonates with a prior investigation, which uncovered a significant inverse correlation between job satisfaction and burnout among ATCs (8). This suggests that ATCs with higher job satisfaction tend to experience lower levels of burnout. According to the Conservation of Resources Theory, job satisfaction serves as a psychological resource that enables individuals to effectively cope with work-related stress (33). When employees are content with their work environment, compensation, or interpersonal relationships, their psychological resources become more plentiful, thus alleviating the burnout resulting from resource depletion (34). For example, a higher level of job satisfaction may augment positive emotions toward work among ATCs.

As is evident in Table 5 and Figure 2, job satisfaction positively affects safety behavior. To be specifically, as shown in Table 4, work itself, work environment and workgroups serve as robust predictors of safety participation, and workgroups play a pivotal role in determining safety compliance. ATCs who experienced a greater degree of job satisfaction are more likely to be motivated and proactive in fulfilling their duties. They allocate more time and exert more effort in task completion, leading to enhanced job performance. A previous study indicated that ATCs with higher levels of job satisfaction were more predisposed to offer assistance to their colleagues, thus contributing to the optimization of workload within high-demand teams at the group level (5), which aligns with H3. Otherwise, this research uncovered a pronounced negative effect of burnout on safety behavior among ATCs, thus lending support to Hypothesis H2. The high levels of work stress exposure experienced by ATCs may exert a dual negative effect on both job satisfaction and burnout (35). However, group cohesion can mitigate the adverse impacts caused by stress exposure (36). Therefore, strengthening group cohesion among ATCs could serve as an effective approach to enhancing their job satisfaction and reducing burnout.

Hypothesis H4 also receives support: burnout among ATCs is a mediating variable in the relationship between job satisfaction and safety behavior. Our findings are consistent with previous research that investigated a similar model in other fields (37, 38). According to social exchange theory, employees engage in positive behaviors at work in exchange for resources and support provided by the organization (39). Job satisfaction can be regarded as an emotional reaction to the exchange relationship between employees and the organization. Conversely, burnout occurs when employees sense an imbalance or unfairness in this exchange relationship, which gives rise to negative attitudes and emotions toward work. This study addresses a gap in the existing literature on the safety behavior of ATCs by comprehensively investigating the mediating function of burnout between job satisfaction and safety behavior.

### 4.2 Feature importance analysis of ATCs’ safety behavior

In order to address Research Question 2, decision tree models were employed. This approach was used to identify the key factors influencing ATC safety behavior. The analysis revealed that burnout emerged as the most critical predictor, followed by job satisfaction, position and post. In contrast, marital status and working year exhibited zero feature importance, suggesting these variables lack significant influence on safety behavior outcomes. These findings align with prior research emphasizing psychological states and organizational factors as determinants of workplace safety (5, 6). The use of decision tree regression and MSE-based feature importance quantification provided a transparent framework for evaluating feature importance in a complex dataset. Overall, the results not only corroborate the findings on the mediating effects identified in the present study, while also highlighting the impact of burnout and job satisfaction on safety behaviors among ATCs.

### 4.3 Prospects and limitations

This study makes a pioneering effort to understand the relationship between job satisfaction and safety behavior within the ATC community, confirming the partially mediating role of burnout. Notably, while most investigations have focused on the direct relationship between job satisfaction and safety behavior, few have examined the mediating role of burnout within the actual work structure of specific occupational groups. Managers and executives should prioritize stress management for ATCs, aiming to mitigate factors contributing to low job satisfaction and implement proactive strategies to manage this high-risk burnout population. The findings of this study have significant implications for the Civil Aviation Administration of China (CAAC) and ATCs.

However, this study has several limitations. First, the application of bootstrapping with a relatively small sample size may compromise the stability of the results. Second, beyond job satisfaction and burnout, other factors potentially influencing ATCs’ safety behavior—such as job stress, psychological capital, and family–work conflict—were not included in this investigation; future research should explore these dimensions. Subsequent studies could further examine how these additional factors influence ATCs’ safety behavior. Finally, as all participants were drawn exclusively from China, the generalizability of our findings to ATCs’ in other cultural contexts may be limited. Therefore, extrapolation of these conclusions requires caution.

## Data Availability

The raw data supporting the conclusions of this article will be made available by the authors, without undue reservation.

## Supplementary material

Supplementary FILE 1 The dataset used in this study.

Supplementary FILE 2 The code for the decision tree model used in the data analysis of this study.
