# Supplementary material for: Job satisfaction, burnout, and safety behavior in air traffic controllers: a mediation analysis and decision tree insights
Source: Front Public Health. 2025 Sep 12;13:1626328. doi: 10.3389/fpubh.2025.1626328 (PMC12464003; doi:10.3389/fpubh.2025.1626328)
Supplement: Supplementary FILE 2 — The code for the decision tree model used in the data analysis of this study. [file Table_2.DOCX]

import pandas as pd
import numpy as np
import matplotlib.pyplot as plt
from sklearn.tree import DecisionTreeRegressor, plot_tree
from sklearn.model_selection import train_test_split, RandomizedSearchCV
from sklearn.metrics import r2_score, mean_squared_error
from sklearn.pipeline import Pipeline


# 自定义伪R²计算函数（基于Fokkema等人定义）
def pseudo_r2(y_true, y_pred):
 *"""计算伪R²（适用于回归问题）
 Args:
 y_true (array): 真实值
 y_pred (array): 预测值
 Returns:
 float: 伪R²值
 """* brier_score = mean_squared_error(y_true, y_pred)
 y_var = np.var(y_true)
 return 1 - (brier_score / y_var)


# 数据加载函数
def data_loader(path):
 *"""加载Excel数据并分割特征与目标
 Args:
 path (str): Excel文件路径
 Returns:
 X_train, X_test, y_train, y_test (tuple): 分割后的数据集
 """* try:
 df = pd.read_excel(path)
 X = df.iloc[:, 1:] # 假设第一列为目标变量
 y = df.iloc[:, 0]
 return train_test_split(X, y, test_size=0.2, random_state=33)
 except Exception as e:
 print(f"数据加载失败: {str(e)}")
 raise


# 评估指标生成器（添加伪R²）
def generate_metrics(y_true, y_pred):
 *"""生成模型评估指标
 Returns:
 pd.Series: 包含伪R²、R²、MSE、RMSE的序列
 """* return pd.Series({
 'Pseudo_R2': pseudo_r2(y_true, y_pred), # 新增伪R²
 'R2': r2_score(y_true, y_pred),
 'MSE': mean_squared_error(y_true, y_pred),
 'RMSE': np.sqrt(mean_squared_error(y_true, y_pred))
 })


# 模型超参数配置
PARAM_GRID = {
 'regressor__criterion': ['squared_error', 'friedman_mse', 'absolute_error'],
 'regressor__splitter': ['best', 'random'],
 'regressor__max_depth': [None, 5, 7, 9, 11, 15],
 'regressor__min_samples_split': np.linspace(0.01, 0.1, 5),
 'regressor__min_samples_leaf': np.linspace(0.005, 0.05, 5),
 'regressor__max_features': ['sqrt', 'log2', 0.7, 0.8, None],
 'regressor__ccp_alpha': [0.0, 0.01, 0.02],
 'regressor__max_leaf_nodes': [None, 20, 40, 60],
 'regressor__min_impurity_decrease': [0.0, 0.01, 0.05]
}


# 主建模函数
def enhanced_modeling(data_path):
 *"""执行完整建模流程
 Returns:
 report_data (DataFrame): 性能报告
 feature_importance (Series): 特征重要性排序
 """* # 数据加载
 X_train, X_test, y_train, y_test = data_loader(data_path)

 # 计算目标变量的方差（用于伪R²计算）
 y_var = np.var(y_test)
 print(f"目标变量方差: {y_var:.4f}")

 # 创建模型管道
 model_pipeline = Pipeline([
 ('regressor', DecisionTreeRegressor(random_state=30))
 ])

 # 超参数优化 - 使用伪R²作为唯一参照指标
 grid_search = RandomizedSearchCV(
 estimator=model_pipeline,
 param_distributions=PARAM_GRID,
 n_iter=100,
 scoring='neg_mean_squared_error', # 使用MSE作为代理指标
 refit=True, # 需要refit以获取完整模型
 cv=5,
 n_jobs=-1,
 verbose=1,
 random_state=4
 ).fit(X_train, y_train)

 # 获取所有候选模型的伪R²评分
 results = pd.DataFrame(grid_search.cv_results_)
 results['pseudo_r2'] = 1 - (results['mean_test_score'].abs() / y_var)

 # 找到伪R²最高的模型
 best_idx = results['pseudo_r2'].idxmax()
 best_pseudo_r2 = results.loc[best_idx, 'pseudo_r2']
 best_params = results.loc[best_idx, 'params']

 print(f"\n最优伪R²: {best_pseudo_r2:.4f}")
 print("最优参数:", best_params)

 # 使用最优参数重新训练模型
 final_model = model_pipeline.set_params(**best_params).fit(X_train, y_train)
 dt_model = final_model.named_steps['regressor']

 # 特征重要性分析
 feature_importances = pd.Series(
 dt_model.feature_importances_,
 index=X_train.columns,
 name='Importance'
 )
 sorted_importances = feature_importances.sort_values(ascending=False)

 # 可视化特征重要性
 plt.figure(figsize=(10, 6))
 plt.rcParams['font.family'] = 'serif' # 使用衬线字体
 plt.rcParams['font.serif'] = ['Times New Roman'] # 指定Times New Roman
 plt.rcParams['mathtext.fontset'] = 'custom' # 自定义数学字体
 plt.rcParams['mathtext.rm'] = 'Times New Roman' # 常规数学字体
 plt.rcParams['mathtext.it'] = 'Times New Roman:italic' # 斜体
 plt.rcParams['mathtext.bf'] = 'Times New Roman:bold'
 sorted_importances.plot(kind='barh', color='skyblue')
 plt.title('Feature Importance Ranking')
 plt.xlabel('Importance Score')
 plt.tight_layout()
 plt.savefig('feature_importance.png', dpi=300)

 # 生成评估报告
 train_pred = final_model.predict(X_train)
 test_pred = final_model.predict(X_test)

 report_data = pd.DataFrame({
 'Train': generate_metrics(y_train, train_pred),
 'Test': generate_metrics(y_test, test_pred)
 }).T

 # 保存结果
 try:
 sorted_importances.to_csv('feature_importance_ranking.csv', header=True)
 report_data.to_csv('model_performance.csv')
 results.to_csv('cv_results_with_pseudo_r2.csv') # 保存所有CV结果
 except Exception as e:
 print(f"文件保存失败: {str(e)}")

 # 打印特征重要性
 print("\n" + "=" * 50)
 print("特征重要性排序:")
 print(sorted_importances.to_markdown())
 print("=" * 50)

 return report_data, sorted_importances


# 主程序
if __name__ == "__main__":
 DATA_PATH = 'C:/Users/84270/Desktop/学术垃圾/管制员/清洗后数据.xlsx'

 try:
 performance_report, feature_importance = enhanced_modeling(DATA_PATH)

 print("\n模型性能报告:")
 print(performance_report.to_markdown())
 except FileNotFoundError:
 print(f"文件路径不存在: {DATA_PATH}")
 except PermissionError:
 print("文件被其他程序占用，请关闭Excel文件后重试")
 except Exception as e:
 print(f"程序运行异常: {str(e)}")
